# Development of automated region of interest selection algorithms for surface‐guided radiation therapy of breast cancer

**DOI:** 10.1002/acm2.14216

**Published:** 2023-12-20

**Authors:** Songye Cui, Guang Li, Hsiang‐Chi Kuo, Bo Zhao, Anyi Li, Laura I. Cerviño

**Affiliations:** ^1^ Department of Medical Physics Memorial Sloan Kettering Cancer Center New York USA

**Keywords:** automation, breast cancer, surface‐guided radiotherapy

## Abstract

To investigate automation of the preparation of the region of interest (ROI) for surface‐guided radiotherapy (SGRT) of the whole breast with two algorithms based on contour anatomies: using the body contour, and using the breast contour. The patient dataset used for modeling consisted of 39 breast cancer patients previously treated with SGRT. The patient's anatomical structures (body and ipsilateral breast) were retrieved from the planning system, and the clinical ROI (cROI) drawn by the planners was retrieved from the SGRT system for comparison. For the body‐contour‐based algorithm, a convolutional neural network (MobileNet‐v2) was utilized to train a synthetic human model dataset to predict body joint locations. With the body joint location knowledge, an automated ROI (aROI^body^) can be created based on: (1) the superior‐inferior (S‐I) borders defined by the joint locations, (2) the left‐right (L‐R) borders defined with 3/4 of chest width, and (3) a curation of the ROI to avoid the ipsilateral armpit. For the breast‐contour‐based algorithm, an aROI^breast^ was created by first defining the ROI in the S‐I direction with the ipsilateral breast boundaries. Other steps are the same as with the body‐contour‐based algorithm. Among the 39 patients, 24 patients were used to fine‐tune the algorithm parameters, and the remaining 15 patients were used to evaluate the quality of the aROIs against the cROIs. A blinded evaluation was performed by three SGRT expert physicists to rate the acceptability and the quality (1–10 scale) of the aROIs and cROIs, and the dice similarity coefficient (DSC) was also calculated to compare the similarity between the aROIs and cROIs. The results showed that the average acceptability was 14/15 (range: 13/15–15/15) for cROIs, 13.3/15 (range: 13/15–14/15) for aROI^body^, and 14.6/15 (range: 14/15–15/15) for aROI^breast^. The average quality was 7.4 ± 0.8 for cROIs, 8.1 ± 1.2 for aROI^body^, and 8.2 ± 0.9 for aROI^breast^. The DSC with cROIs was 0.81 ± 0.06 for aROI^body^, and 0.83 ± 0.04 for aROI^breast^. The ROI creation time was ∼120 s for clinical, 1.3 s for aROI^body^, and 1.2 s for aROI^breast^. The proposed automated algorithms can improve the ROI compliance with the SGRT protocol, with a shortened preparation time. It is ready to be integrated into the clinical workflow for automated ROI preparation.

## 1 INTRODUCTION

In conventional radiotherapy, radiation therapists need to visually align tattoos or marks on the patient's skin to room lasers in order to set up the patient in the correct treatment position.^[^ ^1^ ^]^ With improved camera technology and computing power, the patient's 3D surface can be digitally reconstructed using a stereo camera system. By registering the real‐time patient's surface with the initial reference surface, the translation and rotation errors from the initial patient setup can be estimated then the patient's position can be corrected following the registration results. This technique is known as surface‐guided radiation therapy (SGRT), where a patient's surface is used as a surrogate of the target for patient setup before treatment and/or patient motion monitoring during treatment.^[^ ^1^ ^]^

According to a recent SGRT survey,^[^ ^2^ ^]^ surface imaging systems have been adopted by an increasing number of clinics over the past decade because of their abilities to perform initial patient positioning and intra‐fraction monitoring without the use of ionizing radiation. Typical applications of these systems include open‐face mask stereotactic radiosurgery procedures and breast radiotherapy.^[^ ^2^ ^]^ To calculate the real‐time shifts from the initial patient setup during SGRT, therapists, dosimetrists or physicists must define a region of interest (ROI), so a rigid registration algorithm can be performed within this region between the real‐time image and the reference image to evaluate motion.

However, manually defining a ROI is labor intensive. In addition, the training and experience of the preparers may lead to great disparity in the quality of ROI definition, which influences surface imaging registration results as indicated in a recent study.^[^ ^3^ ^]^ With the increase in SGRT use in clinic, the impact of the conventional procedure to define a ROI in SGRT can be magnified and the efficiency of workflows can be reduced.

In this work, we aim to develop, and ultimately to implement, automated ROI drawing algorithms for breast SGRT to improve the ROI compliance with the SGRT protocol and the efficiency of workflows.

## 2 METHODS AND MATERIALS

Two automated ROI (aROI) selection algorithms have been developed for breast SGRT patients: body‐contour‐based (aROI^body^) and breast‐contour‐based (aROI^breast^). In this section, the patient data and clinical protocol for defining an ROI will be described, and these two automated ROI algorithms will be described.

### 2.1 Patient data and clinical protocol to define an ROI

Thirty‐nine breast cancer patients were set up with SGRT using AlignRT systems (VisionRT, London, UK). Each patient received a mono isocentric, whole breast treatment in supine position, with a deep inspiration breath hold technique. The clinical ROI (cROI) used in the setup was retrospectively retrieved from the AlignRT system and the patients' anatomical structures (the external body contour and the delineated breast) were retrieved from the Eclipse planning system (v15.5 Varian, CA). Among these patients, 24 randomly selected patients (non‐survey patients) were used to determine how to set the superior‐inferior (S‐I) boundaries in both aROI algorithms. The remaining 15 patients (survey patients) were selected to evaluate the quality of aROIs.

The cROIs were manually created based on our institutional protocol: set the ROI boundaries (1) from supraclavicular matchline to 2 cm below the breast in the superior‐inferior (S‐I) direction, (2) from ipsilateral body anterior‐posterior (A‐P) midline to the middle of the contralateral breast in the left‐right (L‐R) direction. Finally, the armpit and the shoulder should be excluded from the ROI.

### 2.2 Body‐contour‐based automated ROI algorithm

**FIGURE 1:**
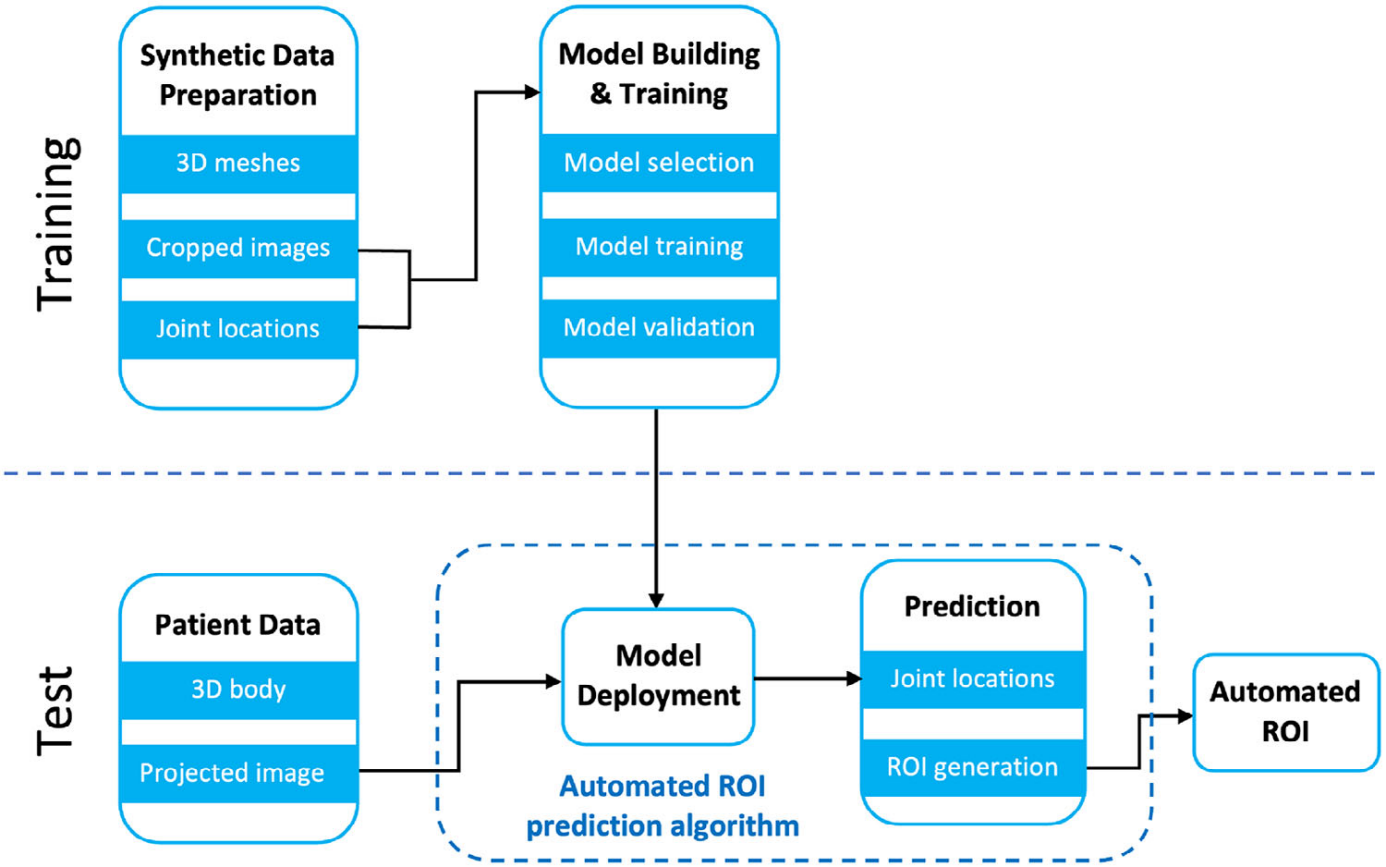
The flowchart of the aROI^body^ algorithm. In the training step, a model was trained with the synthetic data, to predict the joint location. In the test step, the trained model was deployed to generate an automated ROI for the patients. ROI, region of interest.

The flowchart of the aROI^body^ algorithm is shown in Figure 1. We first synthesized a human model dataset that contains 2D body images and body joint locations. In the training step, a deep learning model was trained with the synthetic data to predict joint locations based on the input 2D body image. In the test step, a set of joint locations were predicted for the patients with the deployed trained model, and an aROI was defined using the joints. We elaborate on each step of this approach in the following sections.

#### 2.2.1 Synthetic data preparation

**FIGURE 2:**
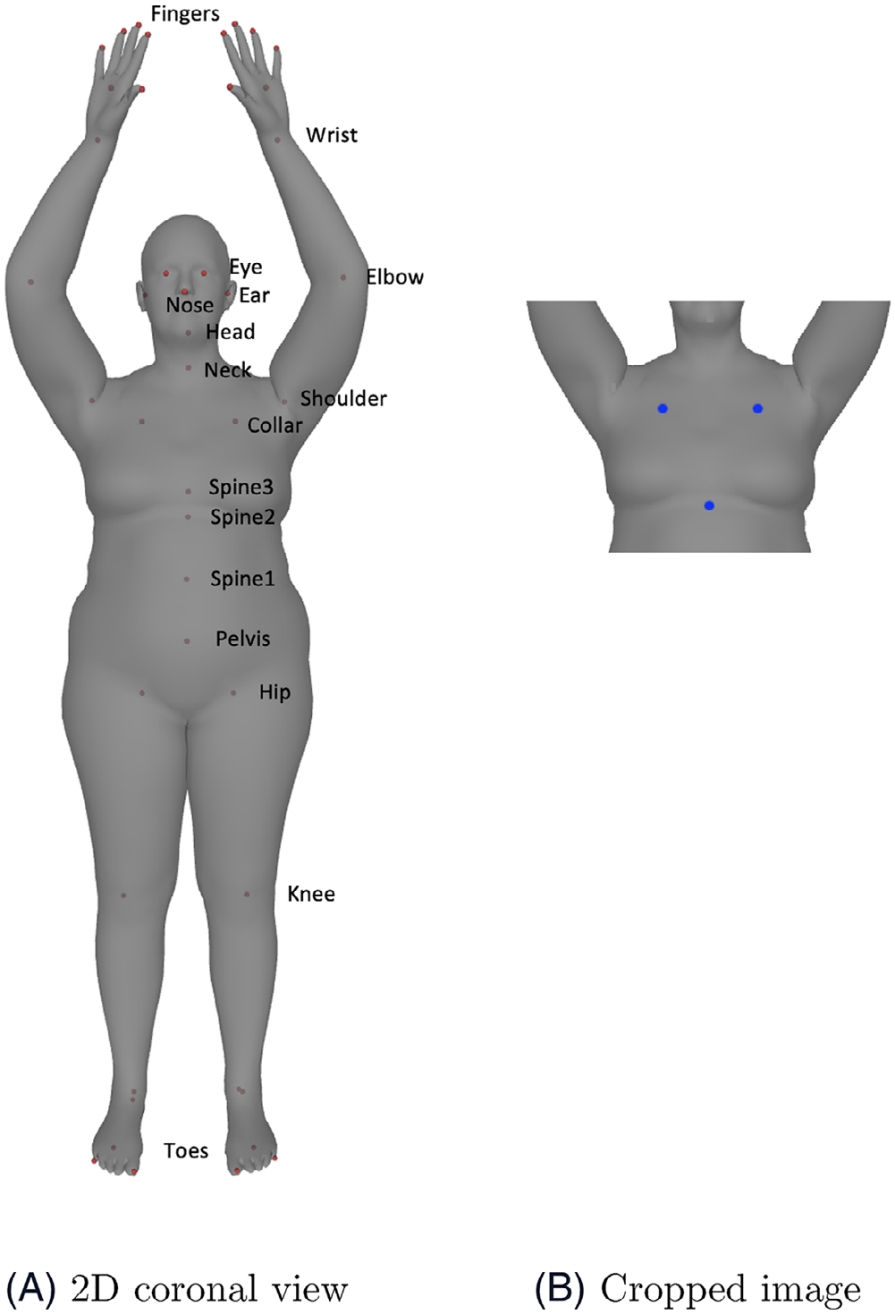
Synthetic data preparation: (A) A 2D coronal view of an SMPL 3D body mesh with annotated joints (ignoring the annotations of joints on the right side for better visualization purposes), (B) A cropped 2D image with joint locations used to train the deep learning model (Left Collar, Right Collar and Spine2 in blue dots). SMPL, skinned multi‐person linear.

SMPL (Skinned Multi‐Person Linear)^[^ ^4^ ^]^ is a realistic 3D human body model that was learned from thousands of 3D body scans, and which can capture body shape and pose variation. In this study, the SMPL model was used to create 3000 realistic 3D human body meshes and the corresponding skeletons (estimated joint locations). The shape of the body meshes was randomly initialized, and the pose of the body meshes was fixed to mimic the pose of breast SGRT patients whose arms are raised as shown in Figure 2a.

For each mesh, and to simplify computation, a 2D image was first generated by projecting the 3D mesh in the coronal view. To match the CT scan range of breast SGRT patients, the full body image was cropped from the upper boundary (randomly between the nose and head joints in Figure 2a) to the lower boundary (randomly between Spine2 and Spine1 joints in Figure 2a).

The synthetic dataset contains 3000 images (size: 200×200×1), along with the corresponding ground truth of joint locations, as shown in Figure 2b. The dataset was split into the training and validation dataset with an 85:15 ratio for further usage.

#### 2.2.2 Model building and training

The MobileNetV2 model^[^ ^5^ ^]^ pretrained on the ImageNet dataset^[^ ^6^ ^]^ was used as a backbone through a transfer learning technique. As described in the reference,^[^ ^5^ ^]^ the architecture of MobileNetV2 contains the initial fully convolution layer with 32 filters, followed by 19 residual bottleneck layers. Dropout and batch normalization were also utilized during training. Details of the architecture can be found in the literature.^[^ ^5^ ^]^ The model was trained with our synthetic dataset: the input of 2D cropped gray‐scale images of a human model, and the output of the model consists of three joint locations (i.e., Left Collar, Right Collar, and Spine2, blue dots in Figure 2b). During the training, the performance of the model was evaluated using Adam optimizer under a stopping criteria of 2000 epochs and a loss function of the Mean Squared Error (MSE) between the ground truth and the predicted joint locations.

#### 2.2.3 ROI generation

**FIGURE 3:**
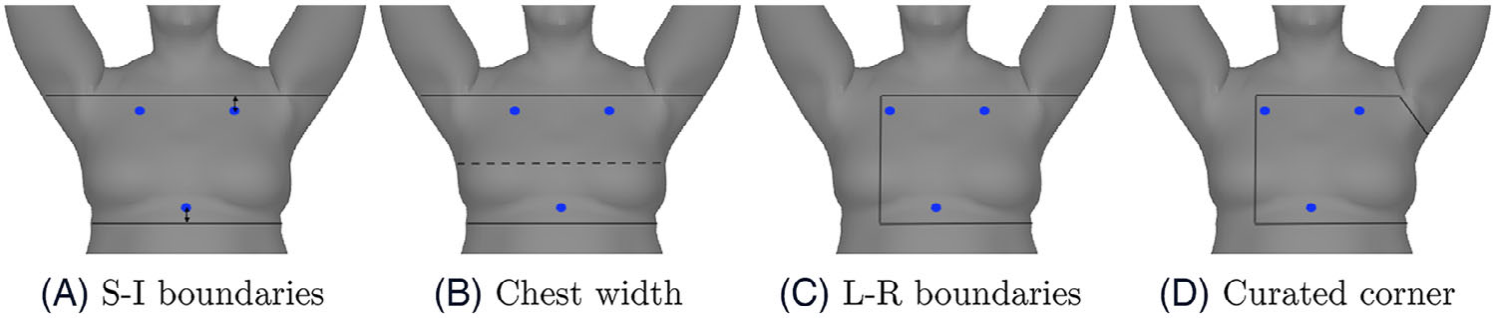
Four steps to generate a ROI from the predicted joints (blue dots): (A) define ROI boundaries in S‐I direction, by extending from the predicted joints, (B) define the chest width using the midline of the upper and lower boundaries, (C) define the boundary in L‐R direction with 3/4 of the chest width, (D) curate the ipsilateral corner to avoid the armpit. L‐R, left‐right; ROI, region of interest; S‐I, superior‐inferior.

We hypothesize that the ROI for breast SGRT patients can be defined from the joint locations. Therefore, following the model deployment, the predicted joints were used to create an ROI. This process is based on a four‐step approach as can be seen in Figure 3. First, the boundaries of the ROI in the S‐I direction were defined by extending upwards from the predicted collar joints and downwards from the predicted Spine2 joint (the optimal extension amount will be determined Section 2.4.1). Second, the chest width was defined with the midline of the upper and the lower boundaries. Third, the boundary of the ROI in the L‐R direction was defined with 3/4 of chest width. Finally, the ipsilateral corner was curated with an arbitrary retraction distance of 80 mm in L‐R and S‐I directions to avoid the armpit. The choice of an 80 mm retraction distance was based on the average size and anatomical considerations observed across this patient cohort. Specifically, the cohort's height ranged from 149.3 to 160.6 cm, and weight varied between 47.7 to 123 kg.

### 2.3 Breast‐contour‐based automated ROI algorithm

**FIGURE 4:**
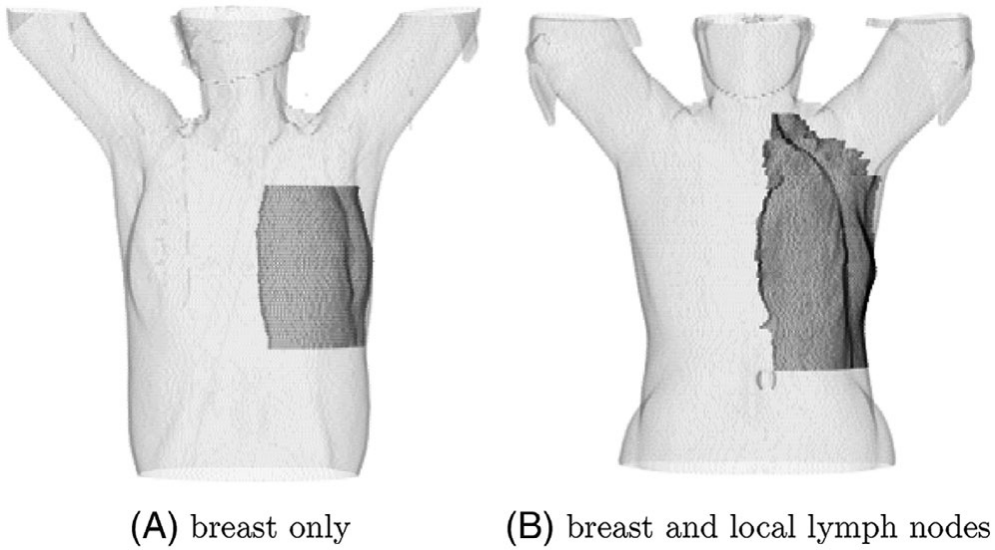
Two types of ipsilateral breast contours delineated at our institute: (A) breast_eval (breast only) and (B) Planning Target Volume(PTV) (breast and local lymph nodes).

As the ipsilateral breast could be delineated differently depending on if local lymph nodes were included, the patient dataset was further categorized into two groups: breast_eval (breast only, 30 out of 39 patients in this group) in Figure 4a, and Planning Target Volume(PTV) (breast and local lymph nodes, 9 out of 39 patients in this group) in Figure 4b.

**FIGURE 5:**
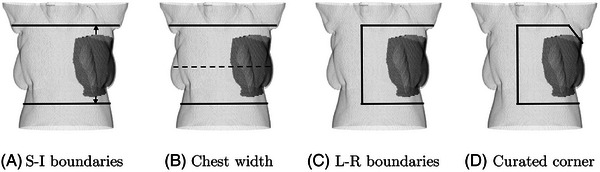
Four steps to generate an ROI from the delineated ipsilateral breast contour (black dots in dark shade): (A) define the ROI boundaries in S‐I direction based on the breast contour, (B) define the chest width using the midline of the upper and lower boundaries, (C) define the boundary in L‐R direction with 3/4 of the chest width, (D) curate the ipsilateral corner to avoid the armpit. L‐R, left‐right; ROI, region of interest; S‐I, superior‐inferior.

Figure 5 illustrates the flowchart of the aROI^breast^ algorithm. This flowchart is similar to the one for aROIbody generation approach (Section 2.2.3). However, in the first step, the extension to form aROI^breast^ is based on the boundaries of breast contour in the S‐I direction, instead of the predicted joint locations. The extension amount is different for each type of the breast contour (Section 3.2.1).

### 2.4 Refinement of ROI algorithms

#### 2.4.1 Body‐contour‐based automated ROI algorithm

To evaluate the accuracy of the predicted joint locations over the validation set of the synthetic data, the Mean Per Joint Position Error (MPJPE) was calculated. MPJPE is defined as the Euclidean distance between ground truth and prediction for a joint.^[^ ^7^ ^]^ Furthermore, to convert the unitless SMPL human model into cm, the height of the model was scaled to the average US female adult height (161.7 cm) reported by the Centers for Disease Control and Prevention (CDC).^[^ ^8^ ^]^

We fine‐tuned various extension amounts (0 to 45 mm with a spacing of 5 mm), which was determined to achieve an optimal dice similarity coefficient (DSC) result between the aROI^body^ and the cROIs over the non‐survey patients. The results were used to create aROI^body^ for the 15 survey patients.

#### 2.4.2 Breast‐contour‐based automated ROI algorithm

For each category of delineated breast, the average distance between the boundaries of clinical ROI and the breast (in S‐I direction) was calculated over the 24 non‐survey patients. The values were used to create aROI^breast^ for the 15 survey patients.

### 2.5 Evaluation of automated ROI algorithms

The remaining 15 cases from the patient cohort were used to evaluate the quality of aROIs (aROI^body^ and aROI^breast^). Qualitatively, a blinded evaluation was performed by three SGRT physicists to rate the acceptability and the quality (1–10 scale, with 10 being the best). Acceptability indicates whether these ROIs can comply with our institutional protocol or not. Quality rating indicates how these ROIs comply with our institutional protocol. Quantitatively, dice similarity coefficient (DSC) was calculated to compare the similarity between the cROI and aROIs. Finally, the computing time for each method was recorded and compared. The computations of the aROIs were performed with an Intel CPU (Xeon Gold 6230, 2.1 GHz, 27.5 MB).

## 3 RESULTS

### 3.1 Body‐contour‐based automated ROI algorithm

#### 3.1.1 Joint location prediction

The average Mean Per Joint Position Error (MPJPE) is 0.22 ± 0.18 cm in the S‐I direction, and 0.37 ± 0.74 cm in the L‐R direction.

#### 3.1.2 Determination of the shifts from the predicted joints to ROI coverage

**FIGURE 6:**
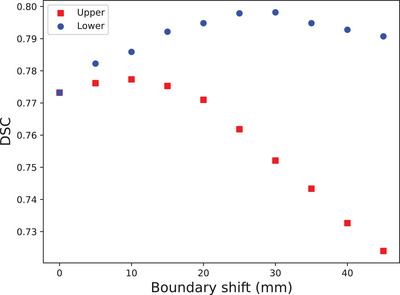
The effects of the shifts in the upper/lower boundary on the DSC results between the automated ROIs and the clinical ROIs. DSC, dice similarity coefficient; ROI, region of interest.

As shown in Figure 6, to achieve an optimal DSC result between the aROI^body^ and the cROIs, a 10 mm shift in the upper boundary and a 30 mm shift in the lower boundary should be applied to extend to an ROI from the predicted joints.

### 3.2 Breast‐contour‐based automated ROI algorithm

#### 3.2.1 Determination of the extension from the breast boundaries to ROI coverage

For the breast_eval category (breast only), a similar process to the one depicted in Figure 6 was carried out, and it was determined that the optimal distance between the boundaries of the breast and the ROI is 27 mm (superior) and 12 mm (inferior). For the PTV category (breast and local lymph nodes), the optimal distance between the boundaries of the breast and the ROI is −24 mm (superior) and 17 mm (inferior).

### 3.3 Comparison between clinical ROI and two automated ROIs

**FIGURE 7:**
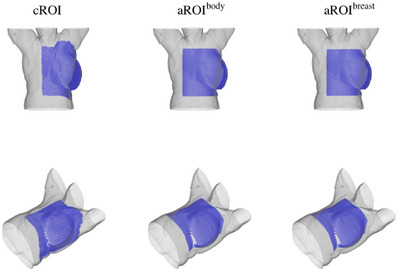
An example of the survey form to compare the clinical ROIs and two types of automated ROIs. ROI, region of interest.

An example of the survey ROI presentation is shown in Figure 7. The overall performance of the blinded evaluation result is shown in Table 1. The average quality rating was 7.4 ± 0.8 for cROIs, 8.1 ± 1.2 for aROI^body^, and 8.2 ± 0.9 for aROI^breast^, respectively. The average acceptability was 14/15 (range: 13/15–15/15) for cROIs, 13.3/15 (range: 13/15–14/15) for aROI^body^, and 14.6/15 (range: 14/15–15/15) for aROI^breast^. The DSC was 0.81 ± 0.06 between the cROIs and the aROI^body^, 0.83± 0.04 between the cROIs and the aROI^breast^, and 0.91 ± 0.08 between the aROI^body^ and the aROI^breast^, respectively.

**TABLE 1:** The evaluation results of ROIs over the 15 cases.

				Quality			Acceptability		
ID	Age	L/R	Vol (cm^3^)	cROI	aROI^body^	aROI^breast^	cROI	aROI^body^	aROI^breast^
1	62	L	1358	8.7	5.3	7.7	3/3	1/3	3/3
2	83	R	1017	8.3	9.3	7.3	3/3	3/3	3/3
3	88	L	684	7.7	5.3	9.3	3/3	1/3	3/3
4	73	L	1863	7.7	9.0	6.7	3/3	3/3	3/3
5	73	L	1295	6.0	7.7	9.0	2/3	3/3	3/3
6	78	L	820	7.7	9.0	6.7	3/3	3/3	3/3
7	84	L	1344	7.0	8.3	8.7	3/3	3/3	3/3
8	65	L	1461	7.0	8.7	8.3	2/3	3/3	3/3
9	73	L	1039	8.0	8.3	8.7	3/3	3/3	3/3
10	48	L	1769	7.3	9.0	9.0	3/3	3/3	3/3
11	94	L	406	8.0	8.3	6.3	3/3	2/3	2/3
12	83	L	957	7.3	8.7	9.0	3/3	3/3	3/3
13	74	L	1504	7.7	7.3	9.0	3/3	3/3	3/3
14	69	L	2837	6.7	8.3	8.3	3/3	3/3	3/3
15	74	L	2214	6.0	8.7	7.3	2/3	3/3	3/3
all				7.4 ± 0.8	8.1 ± 1.2	8.2 ± 0.9	14/15	13.3/15	14.6/15

*Note*: Error values are the standard deviation.

Abbreviations: ID, patient number; L/R, left/right of breast laterality; Vol, breast volume; Quality, the values are the average quality rating of three evaluators; Acceptability, in format of *n*/3, means *n* out of 3 evaluators consider the ROI as acceptable.

According to the feedback given by the three SGRT physicists following the survey, the most common reason for the unacceptable ROIs is too much coverage of the movable arm/armpit. Other reasons include inadequate coverage of the breast, and too much beyond the superior or the inferior side. To correct the unacceptable ROIs, only minor edits (border trimming) are needed.

Based on the observations, the time to define a cROI is around 2 min. In comparison, the average time is 1.34 s to create an aROI^body^, and 1.2 s to create an aROI^breast^. An extra time (1.6 s) to load the trained model is needed for the aROI^body^ algorithm.

## 4 DISCUSSION

The implementation of automation in clinical radiation oncology usually includes image registration, treatment planning, plan evaluation, automation of QA/QC tasks.^[^ ^9^ ^]^ However, the implementation of automation in SGRT is sparse. Chen et al.^[^ ^10^ ^]^ have developed an algorithm that automates the ROI selection in SGRT. This algorithm predicts the registration error by using a Convolutional Neural Network (CNN) to analyze the curvature of the patient's surface anatomy. In our proposed approaches, we eschew reliance on the curvature of the patient's surface for determining the breast region, as it may not yield consistent reliability.

Two automation algorithms have been developed to address two clinic scenarios: when breast contours are provided, and when breast contours are not provided. The aROI^breast^ algorithm would be preferred if the breast contour is available, as the generation of aROI^breast^ better comply to our institutional protocol compared to others (shown in Table 1). One disadvantage of this approach is that the delineation of the breast contour could vary among different institutions, therefore the results of the aROI^breast^ in this study might not be easily transferred to other institutions.

In clinical scenarios where the breast contour is not delineated, the aROI^body^ algorithm can be used. The predicted joint locations determine the upper and lower boundaries (the S‐I direction) of ROI (as shown in Figure 3). As mentioned in Section 3.1.1, the error of predicted joint locations is small (0.22 cm) in the S‐I direction, which means our algorithm can confidently predict the joint location. The error in the L‐R direction is relatively large, which means the predicted joint location is more likely to deviate from the ground truth in the L‐R direction, but this does not affect the aROIs in the current algorithm. The advantage of the aROI^body^ algorithm is that the delineation of the breast contour is not required in the treatment plan, and therefore it may be more generic.

Traditionally, it would be straightforward to feed a large amount of clinical data to train a deep learning model.^[^ ^11^ ^]^ It will be time‐consuming to curate the highly user dependent cROIs and prepare a large consistent clinical dataset. Fortunately, such effort is not necessary, as demonstrated in this study, as we were able to use the SMPL^[^ ^4^ ^]^ model to synthesize a set of 3D human model data coupled with joint locations.

The results show cROIs comply with internal SGRT protocol the least, even though they were created under the guidance of it. This is probably because the planners do not have the ruler to make the exact same amount of extension from certain landmarks (either the breast contour or the joint locations).

In the future, the proposed algorithms can be integrated into the current clinical task at our institute. Furthermore, these algorithms could potentially be adapted and used for other treatment sites including head and neck cases.

## 5 CONCLUSION

We have developed a simple algorithm to automate ROI selection for SGRT of breast treatments when breast contours are provided, and a machine learning based algorithm when breast contours are not provided or are not part of the clinical workflow. It was found on average the automated ROI better complies with our institutional protocol than clinical ROI. Automated ROI created based on breast PTV contours best complies with our institutional protocol. The automatic generation took only a small fraction of time compared with manual work. The proposed algorithm is particularly useful in the clinic because it can potentially improve the work efficiency, consistency, and quality of defining ROIs in SGRT.

## AUTHOR CONTRIBUTIONS

Songye Cui performed the experiments, the statistical analysis, and drafted the manuscript.Guang Li, Anyi Li and Laura I. Cerviño contributed to design of the study and review of analysis. Guang Li, Hsiang‐Chi Kuo, Bo Zhao, and Laura I. Cerviño reviewed the manuscript.

## CONFLICT OF INTEREST STATEMENT

The authors declare no conflicts of interest.

## References

[1] Hoisak JDP , Paxton AB , Waghorn BJ , Pawlicki T . Surface Guided Radiation Therapy. CRC Press; 2020.

[2] Padilla L , Havnen‐Smith A , Cerviño L , Al‐Hallaq HA . A survey of surface imaging use in radiation oncology in the United States. J Appl Clin Med Phys. 2019;20:70‐77. 10.1002/acm2.12762 PMC6909172 31743588

[3] Sauer T‐O , Ott OJ , Lahmer G , Fietkau R , Bert C . Region of interest optimization for surface guided radiation therapy of breast cancer. J Appl Clin Med Phys. 2021;22:152‐160. 34543500 10.1002/acm2.13410 PMC8504613

[4] Loper M , Mahmood N , Romero J , Pons‐Moll G , Black MJ . SMPL: a skinned multi‐person linear model. ACM Trans Graph . 2015;34:1‐16.

[5] Sandler M , Howard A , Zhu M , Zhmoginov A , Chen L‐C . MobileNetV2: inverted residuals and linear bottlenecks. In: Proceedings of the IEEE Conference on Computer Vision and Pattern Recognition (CVPR) . IEEE; 2018.

[6] Deng J , Dong W , Socher R , Li L‐J , Li K , Fei‐Fei L . ImageNet: a large‐scale hierarchical image database. In: 2009 IEEE Conference on Computer Vision and Pattern Recognition . IEEE; 2009:248‐255.

[7] Dang Q , Yin J , Wang B , Zheng W . Deep learning based 2D human pose estimation: a survey. Tsinghua Sci Technol. 2019;24:663‐676.

[8] Fryar CD , Kruszan‐Moran D , Gu Q , Ogden CL . Mean body weight, weight, waist circumference, and body mass index among adults : United States, 1999–2000 through 2015–2016. Natl Health Stat Report. 2018;(122):1‐16. 30707668

[9] Moore KL , Kagadis GC , McNutt TR , Moiseenko V , Mutic S . Vision 20/20: automation and advanced computing in clinical radiation oncology. Med Phys. 2014;41:010901. 24387492 10.1118/1.4842515

[10] Chen H , Chen M , Lu W , et al. Deep‐learning based surface region selection for deep inspiration breath hold (DIBH) monitoring in left breast cancer radiotherapy. Phys Med Biol. 2018;63:245013. 30523967 10.1088/1361-6560/aaf0d6

[11] Zhang A , Xing L , Zou J , Wu JC . Shifting machine learning for healthcare from development to deployment and from models to data. Nat Biomed Eng. 2022;6:1330‐1345. 35788685 10.1038/s41551-022-00898-y PMC12063568

